# Mimicking the Biological Sense of Taste In Vitro Using a Taste Organoids‐on‐a‐Chip System

**DOI:** 10.1002/advs.202206101

**Published:** 2023-01-13

**Authors:** Jianguo Wu, Changming Chen, Chunlian Qin, Yihong Li, Nan Jiang, Qunchen Yuan, Yan Duan, Mengxue Liu, Xinwei Wei, Yiqun Yu, Liujing Zhuang, Ping Wang

**Affiliations:** ^1^ Biosensor National Special Laboratory Key Laboratory for Biomedical Engineering of Education Ministry Department of Biomedical Engineering Zhejiang University Hangzhou 310027 P. R. China; ^2^ The MOE Frontier Science Center for Brain Science and Brain‐Machine Integration Zhejiang University Hangzhou 310027 P. R. China; ^3^ State Key Laboratory of Transducer Technology Chinese Academy of Sciences Shanghai 200050 P. R. China; ^4^ College of Life Sciences Zhejiang University Hangzhou 310058 P. R. China; ^5^ Department of Otolaryngology Eye, Ear, Nose and Throat Hospital Shanghai Key Clinical Disciplines of Otorhinolaryngology Fudan University Shanghai 200031 P. R. China; ^6^ Cancer Center Zhejiang University Hangzhou 310058 P. R. China

**Keywords:** bioelectronic tongue, extracellular potential recording, organoid‐on‐a‐chip, taste organoid, taste recognition

## Abstract

Thanks to the gustatory system, humans can experience the flavors in foods and drinks while avoiding the intake of some harmful substances. Although great advances in the fields of biotechnology, microfluidics, and nanotechnologies have been made in recent years, this astonishing recognition system can hardly be replaced by any artificial sensors designed so far. Here, taste organoids are coupled with an extracellular potential sensor array to form a novel bioelectronic organoid and developed a taste organoids‐on‐a‐chip system (TOS) for highly mimicking the biological sense of taste ex vivo with high stability and repeatability. The taste organoids maintain key taste receptors expression after the third passage and high cell viability during 7 days of on‐chip culture. Most importantly, the TOS not only distinguishs sour, sweet, bitter, and salt stimuli with great specificity, but also recognizes varying concentrations of the stimuli through an analytical method based on the extraction of signal features and principal component analysis. It is hoped that this bioelectronic tongue can facilitate studies in food quality controls, disease modelling, and drug screening.

## Graphical abstract

The authors designed a novel bioelectronic taste organoid and developed a taste organoid‐on‐a‐chip system for highly mimicking the biological sense of taste in vitro. The system recognizes sour, sweet, bitter, and salt stimuli with great specificity. Compare to previous works, this bioelectronic tongue is advantaged in the ability to reflect real taste information, stability, and repeatability.

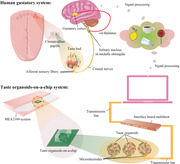

## 1 Introduction

The human gustatory system not only enables us to taste the flavors of foods and drinks but also helps us to avoid the intake of harmful substances.^[^ ^1^ ^]^ In the mammalian gustatory system, taste recognition starts from taste receptor cells which are embedded in the taste buds of tongues.^[^ ^2^ ^]^ These taste receptor cells expressing taste receptors such as OTOP1, T1R2/T1R3, T2Rs, and T1R1/T1R3, can interact with taste stimuli and activate the intracellular signal transduction pathways,^[^ ^3^ ^]^ resulting in the generation of transmembrane currents^[^ ^4^ ^]^ and the transmission of the gustatory information to gustatory axons that entangle them.^[^ ^5^ ^]^ Then, the information was conveyed by cranial nerves to the medulla and finally reached the brain's gustatory cortex for signal processing.^[^ ^6^ ^]^ Basic tastes (sweet, bitter, salty, umami, and sour) are sensed by different taste receptor cells and mediated by different neurons,^[^ ^5^, ^7^ ^]^ which ensures the specificity and accuracy of the taste information. In addition, the taste receptor cells are reproducible that helps maintain the function of the gustatory system for a lifetime. Although great advances in the fields of biotechnology, microfluidics and nanotechnologies have been made in recent years, this astonishing recognition system can hardly be replaced by any artificial sensors designed so far.

Traditional electronic tongues (ETs) mainly use nonspecific, low‐selective, chemical sensors with cross‐sensitivity to different substances in solution and characterize a sample as a whole.^[^ ^8^ ^]^ Although they can recognize samples through pattern recognition methods with high stability, they lack specificity and can hardly reflect taste information from human perspectives. To further improve the performance of ETs, taste receptors, enzymes, molecularly imprinted polymers, and peptides were used in taste recognition.^[^ ^9^ ^]^ Nevertheless, these non‐cell biorecognition elements have no intracellular signal processing and intercellular communications which are vital in the mammalian taste sensing process.^[^ ^10^ ^]^ In the last decade, part of the mammalian gustatory system or the system as a whole has been used as biorecognition elements in bioelectronic tongues for mimicking the biological senses of taste.^[^ ^11^ ^]^ For example, lingual epithelium and its derived primary cells containing taste receptor cells that are responsible for primary taste recognition are used for sensing tastant molecules based on a microelectrode array or a light‐addressable potentiometric sensor.^[^ ^11^, ^12^ ^]^ These bioelectronic tongues demonstrate promising usages in the food industry and drug discovery. However, both lingual epithelium and its derived primary taste receptor cells have low lifespans and suffer from individual variabilities, which greatly impact their practicability and stability.

Organoids are miniaturized organ models formed in vitro using pluripotent stem cells or adult stem cells.^[^ ^13^ ^]^ They highly resemble the native organ in terms of gene and protein expression, metabolic function, and microscale tissue architecture.^[^ ^14^ ^]^ More importantly, the extracellular niche used for organoid culture mimicking the in vivo environment allows the stem cells in the organoid to constantly renew themselves while maintaining their capability to differentiate into the multiple cell types of their tissue of origin.^[^ ^15^ ^]^ The taste organoids are first established by Ren et al.^[^ ^16^ ^]^ They found that taste stem/progenitor cells that expressed Lgr5 or Lgr6 in taste buds self‐assembled in vitro to form spherical structures and differentiated into taste bud cells, including type II and type III taste receptor cells (TRCs).

In this study, to mimic the biological sense of taste ex vivo with high stability and repeatability, we developed a novel bioelectronic tongue based on a bioelectronic organoid (taste organoids‐on‐a‐chip) consisting of organoids formed by taste progenitor cells and a microelectrode array (MEA), namely taste organoids‐on‐a‐chip system (TOS). In the TOS, the MEA functioned as gustatory axons to receive gustatory information by real‐time recording the extracellular potentials of on‐chip taste organoids. Thanks to the self‐renewal activity of taste progenitor cells and the ability to differentiate into different TRCs, the taste organoids maintained key taste receptors expression after the third passage and maintained high cell viability during 7 days of on‐chip culture. More importantly, the system distinguished sour, sweet, bitter, and salt stimuli based on the specificity of individual TRCs to recognize only one type of taste. In addition, through analysis of the differences in characteristic values extracted from the extracellular potentials of the taste organoids, the TOS recognized varying degrees of sour, sweet, bitter, and salty in a biological way which partly reconstituted mammalian taste in vitro.

## 2 Results

### 2.1 Characterization of the Taste Organoids

**Figure 1:**
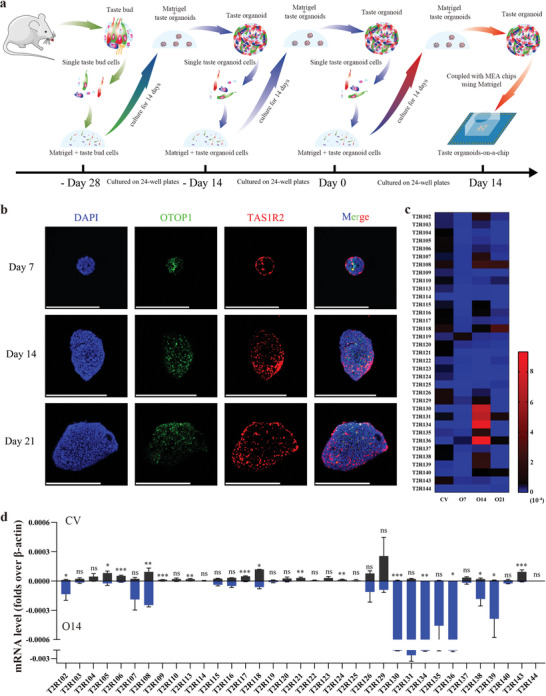
Characterization of taste receptors in circumvallate papillae and taste organoids. a) Schematic illustration of the preparation of the taste organoids‐on‐a‐chip for the TOS set‐up. b) Immunostaining images showing the distribution of OTOP1 and TAS1R2 on the taste organoid cultured for 7, 14, or 21 days. Scale bar = 200 µm. c) mRNA level of T2Rs (mean fold over *β*‐actin) in CV and the taste organoid cultured for 7 (O7), 14 (O14), or 21 days (O21) (*n* = 3). d) Comparison of mRNA level of T2Rs between circumvallate papillae and the taste organoids cultured for 7, 14, or 21 days (*n* = 3). Data are presented as Mean ± SEM. * indicates *p* < 0.05, and ** indicates *p* < 0.01.

The cells extracted from circumvallate papillae were cultured in Matrigel for one generation to screen taste stem/progenitor cells (**Figure** **1**a). The third generation of the cells was used to form organoids for setting up the taste organoids‐on‐a‐chip system. Before the set‐up, we characterized the taste receptor cells in the taste organoids. OTOP1 (Otopetrin‐1) is an ion channel considered as the mammalian sour taste receptor in type III taste receptor cells,^[^ ^7^ ^]^ while TAS1R2 (Taste receptor type 1 member 2) is a component of the main taste receptor for sweetness and is presented in type II taste receptor cells.^[^ ^20^ ^]^ Therefore, we performed immunostaining to visualize these two functional proteins in the taste organoids cultured in a 24‐well plate for 7, 14, and 21 days. Numerous cells are immunopositive for OTOP1 (green) or TAS1R2 (red) in the taste organoids cultured for 7, 14, and 21 days, suggesting that a part of taste stem/progenitor cells in the taste organoids differentiated into type III and II taste receptor cells (Figure 1b). In addition, RT‐qPCR was used to evaluate the mRNA level of 35 bitter taste receptors (T2Rs) in the circumvallate papillae (CV) and the taste organoids (Figure 1c). The expression of most T2Rs (23 in 35 kinds of T2Rs) on mRNA level, reached their peaks on day 14. By contrast, only a few T2Rs’ mRNA expression peaked on days 7 and 21 (4 and 5 kinds of T2Rs, respectively). Therefore, 14 days was chosen as the optimized culture time for the establishment of the taste organoids‐on‐a‐chip system.

Under the optimized culture time, most T2Rs in the taste organoids showed no significant differences (19 T2Rs) compared to the CV in the mRNA expression (Figure 1d). Seven T2Rs were significantly higher expressed in the taste organoids than in the CV, while 9 T2Rs had a significantly lower mRNA level compared to the CV. The difference in the mRNA expression may be a result of the difference between in vivo and in vitro culture conditions, but both immunostaining and RT‐qPCR results suggested the taste organoids cultured at the optimized time of 14 days expressed multiple types of taste receptors and can be used for further taste recognition.

### 2.2 Working Principle of the Taste Organoids‐on‐a‐Chip System

**Figure 2:**
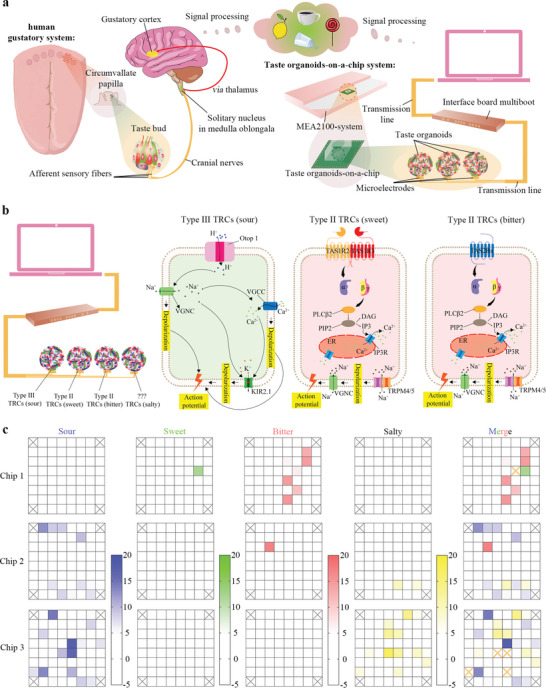
Working principle of the taste organoids‐on‐a‐chip system. a) Comparison of the human gustatory system and taste organoids‐on‐a‐chip system. b) Type III, Type II sweet‐sensing, Type II bitter‐sensing, and as‐yet‐undefined TRCs in the on‐chip taste organoids were able to detect sour, sweet, bitter, and salty stimuli, respectively. The intracellular signal transductions result in the change of intracellular and extracellular ion concentrations and finally result in the action potentials. The changed extracellular ion concentrations can thus be detected by the MEA. c) The extracellular potential PSD changes detected by 64 microelectrodes of three chips exposed to 0.625 g mL^−1^ acetic acid (sour), 0.75 g mL^−1^ sucrose (sweet), 0.125 g mL^−1^ PTC (bitter), and 3 m NaCl (salty). The orange cross means the microelectrode cannot specifically detect taste quality and thus be excluded for further analysis.

The taste organoids‐on‐a‐chip system imitated the structure of the human gustatory system (**Figure** **2**a). The human gustatory sensation starts from taste buds.^[^ ^5^ ^]^ Taste receptor cells in the taste buds are sensitive to taste stimuli and able to transmit taste information to the afferent sensory fibres which entangle them.^[^ ^4^, ^7^, ^21^ ^]^ Then, the taste information reaches the solitary nucleus in the medulla oblongata through cranial nerves and finally be transmitted to the gustatory cortex to form feelings.^[^ ^21^, ^22^ ^]^ Here, the “gustatory sensation” of the taste organoids‐on‐a‐chip system started from the taste organoids. When the on‐chip organoids were exposed to taste stimuli, the taste receptors on the surface of TRCs recognized the stimuli and activated the intracellular signal transduction pathways,^[^ ^3^ ^]^ resulting in the generation of transmembrane currents,^[^ ^4^ ^]^ and therefore the changes of extracellular field potentials^[^ ^23^ ^]^ (Figure 2b). Microelectrodes were coupled with the taste organoids for real‐time recording of their extracellular field potential information. The information was then transmitted to an interface board multiboot and finally reached a computer for signal processing via data wires.

### 2.3 Specificity of the Taste Organoids‐on‐a‐Chip System for Tastants Recognition

In the human gustatory system, the sour, sweet, bitter, and salty stimuli are recognized by different TRCs, as the taste receptors for these four stimuli are always expressed in distinct cell types.^[^ ^3b^ ^]^ Therefore, we suppose that the TOS could distinguish sour, sweet, bitter, and salty stimuli based on the specificities of TRCs in taste organoids. To prove this, the same chip was exposed to sour, sweet, bitter, or salty stimuli for 3 min one after another with an interval of 20 min. Most microelectrodes of the chips that detected obvious changes in PSD of the extracellular potential when exposed to one taste stimuli were found unable to detect obvious changes when exposed to another three stimuli (Figure 2c), suggesting that these microelectrodes were surrounded by only one of the four types of cells. These microelectrodes, therefore, were selected for following specific tastants recognition.

### 2.4 Biocompatibility of the Taste Organoids‐on‐a‐Chip System

**Figure 3:**
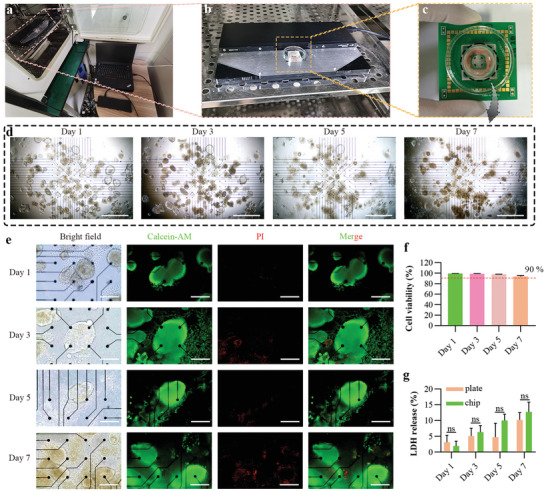
Characterization of biocompatibility of the taste organoids‐on‐a‐chip. a) The overall image of the TOS. b) A MEA2100‐system integrated with a taste organoids‐on‐a‐chip. c) Image of the taste organoids‐on‐a‐chip. d) Bright‐field images of the taste organoids cultured on the chip on day 1, 3, 5, and 7. Scale bar = 1 mm. e) Representative live and dead staining images of the taste organoids cultured on the chip on day 1, 3, 5, and 7. Calcein‐AM (green) was used to label live cells and PI (red) was used to label dead cells. Scale bar = 200 µm f) Cell viability of cells derived from the taste organoids cultured on the chip on day 1, 3, 5, and 7 (*n* = 3). g) Activities of LDH which are released by the taste organoids cultured on the chip or 24‐well plate on day 1, 3, 5, and 7. Data are presented as Mean ± SD. ** indicates *p* < 0.01.

To study the biocompatibility of the TOS, the taste organoids cultured for 14 days were seeded on the microelectrode array (MEA) to construct taste organoids‐on‐a‐chip system (**Figure** **3**a–c) and cultured for 7 days. The bright‐field images showed the sustained growth of the taste organoids on the chip from day 1 to day 7 (Figure 3d), suggesting the good biocompatibility of the taste organoids‐on‐a‐chip system. The good biocompatibility was further supported by the live/dead staining (Figure 3e). The most proportion of cells in the taste organoids after 1, 3, 5, or 7 days’ on‐chip culture were stained by Calcein‐AM (a cell‐permeant dye to label live cells), while only a small proportion of cells were stained by propidium iodide (PI, a nucleic acid dye for labelling dead cells). In addition, the taste organoids were digested into single cells for live/dead staining to precisely calculate the cell viability. During the 7 days of on‐chip culture, cells maintained high cell viability (>90%) (Figure 3f). Furthermore, the lactate dehydrogenase (LDH) cytotoxicity assay data showed that the death percentage of the cells in taste organoids cultured on the chip for 1, 3, 5, and 7 days was 1.91 ± 1.53%, 6.35 ± 2.07%, 10.04 ± 1.96%, and 12.72 ± 3.10%, respectively (Figure 3g). No significant difference was shown in the death percentage between the taste organoids cultured on the chip and the ones cultured on the plates during the 7 days culture. These results suggested that the taste organoids‐on‐a‐chip system was biocompatible and could maintain the high cell viability of the organoid for at least 7 days.

### 2.5 Recognition of Sourness Using the Taste Organoids‐on‐a‐Chip

**Figure 4:**
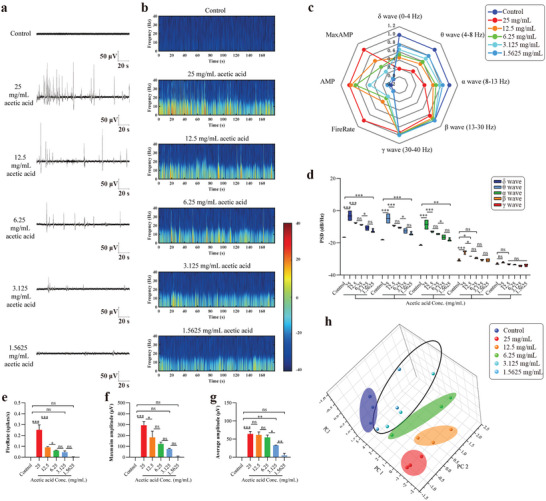
Response of taste organoids‐on‐a‐chip to acetic acid. a) Representative signals of the TOS exposed to 0, 25, 12.5, 6.25, 3.125, and 1.5625 mg mL^−1^ acetic acid. b) Joint time‐frequency analysis of the representative signals. c) Differences in the 8 parameters among the signals of the TOS exposed to the different concentrations of acetic acid. d) Comparison of PSD of different frequency bands among the signals of the TOS exposed to the different concentrations of acetic acid (*n* = 3). e) Fire rates of spikes in the signals of the TOS exposed to the different concentrations of acetic acid (*n* = 3). f) The maximum amplitudes of spikes in the signals of the TOS exposed to the different concentrations of acetic acid (*n* = 3). g) The average amplitudes of spikes in the signals of the TOS exposed to the different concentrations of acetic acid (*n* = 3). h) Classification of the signals based on PCA. Data are presented as Mean ± SD. * indicates *p* < 0.05. ** indicates *p* < 0.01. *** indicates *p* < 0.001.

To study the function of the taste organoids‐on‐a‐chip system in recognition of varying degrees of sourness, the taste organoids were exposed to 25, 12.5, 6.25, 3.125, 1.5625, and 0 mg mL^−1^ acetic acid. Organic acids can permeate type III cells, acidify the cytoplasm, block leak K^+^ channels, and depolarize the cell membrane^[^ ^24^ ^]^, resulting in the change of the extracellular potential. The typical extracellular potentials of the taste organoids exposed to varying concentrations of acetic acid and their energy distributions in 0–40 Hz were shown in **Figure** **4**a and Figure 4b. For analysis of the extracellular potentials, we calculated the power spectral density (PSD) of *δ* (0 – 4 Hz), *θ* (4 – 8 Hz), *α* (8 – 13 Hz), *β* (13 – 30 Hz), and *γ* (30 – 40 Hz) wave in the extracellular potentials. We also measured the fire rate, maximum amplitude, and average amplitude of spikes in the extracellular potentials. Differences in the 8 normalized parameters among varying concentrations of acetic acid were shown in the radar map (Figure 4c). For *δ*, *θ*, and *α* waves, significant higher PSD values were observed in 25, 12.5, 6.25, 3.125, and 1.5625 mg mL^−1^ acetic acid groups, compared to the control group (Figure 4d). For the *β* wave, only 25 and 12.5 mg mL^−1^ acetic acid groups had significant higher PSD values compared to the control group. In contrast, for the *γ* wave, no significant difference was shown between the control and all acetic acid groups. In addition, within the range of 0 – 25 mg mL^−1^, PSD values of *δ*, *θ*, *α*, and *β* waves gradually decreased with the decrease of acetic acid concentration. These results suggested that acetic acid mainly affected the extracellular potential changes in *δ*, *θ*, *α*, and *β* frequency bands.

The fire rate, maximum amplitude, and average amplitude of spikes in the extracellular potentials were also found to decrease with the decline of acetic acid concentration. When exposed to 25 mg mL^−1^ acetic acid, the extracellular potentials of taste organoids changed violently compared to the control, where the fire rate was 0.25 ± 0.08 spikes per s, the maximum amplitude was 293.40 ± 59.83 µV, and the average amplitude was 64.16 ± 11.94 µV. When the concentration of acetic acid went down to 12.5 mg mL^−1^, the fire rate dropped to 0.09 ± 0.01 spikes per s, but from 12.5 to 1.5625 mg mL^−1^, there was a gradual decline in fire rate (0.06 ± 0.01, 0.05 ± 0.02, and 0.001 ± 0.003 spikes per s for 6.25, 3.125, and 1.5625 mg mL^−1^ acetic acid, respectively, Figure 4e). For maximum amplitude, the value went down gradually when the concentration of acetic acid decreased from 25 to 1.5625 mg mL^−1^ (293.40 ± 59.83, 182.90 ± 98.99, 122.50 ± 27.96, 74.91 ± 9.28, and 5.40 ± 9.36 µV for 25, 12.5, 6.25, 3.125, and 1.5625 mg mL^−1^ acetic acid, respectively, Figure 4f). For average amplitude, no significant difference was shown among the value of 25, 12.5, and 6.25 mg mL^−1^ acetic acid groups (64.16 ± 11.94, 61.47 ± 14.61, and 54.45 ± 11.65 µV, respectively), but the value declined sharply when the concentration of acetic acid went down from 6.25 to 1.5625 mg mL^−1^ (32.63 ± 1.240 and 5.40 ± 9.36 µV for 3.125 and 1.5625 mg mL^−1^, respectively, Figure 4g). Finally, according to the result of the principal component analysis based on the 8 parameters, the extracellular potential signals of taste organoids were clustered into 5 types (Figure 4h). In particular, the extracellular potential signals of taste organoids exposed to 1.5625 and 3.125 mg mL^−1^ acetic acid were considered as one type, suggesting that the two concentrations of acetic acid may have a similar taste for taste organoids. Taken together, the results demonstrated that the taste organoids‐on‐a‐chip system had a function in recognizing varying degrees of sourness.

### 2.6 Recognition of Sweetness Using the Taste Organoids‐on‐a‐Chip

**Figure 5:**
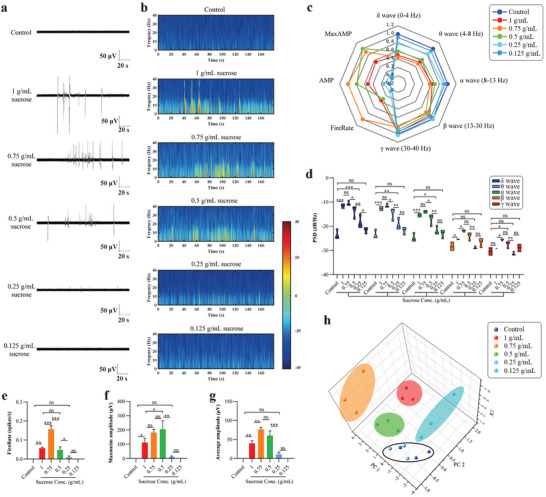
Response of taste organoids‐on‐a‐chip to sucrose. a) Representative signals of the TOS exposed to 0, 1, 0.75, 0.5, 0.25, and 0.125 g mL^−1^ sucrose. b) Joint time‐frequency analysis of the representative signals. c) Differences in the 8 parameters among the signals of the TOS exposed to the different concentrations of sucrose. d) Comparison of PSD of different frequency bands among the signals of the TOS exposed to the different concentrations of sucrose (*n* = 3). e) Fire rates of spikes in the signals of the TOS exposed to the different concentrations of sucrose (*n* = 3). f) The maximum amplitudes of spikes in the signals of the TOS exposed to the different concentrations of sucrose (*n* = 3). g) The average amplitudes of spikes in the signals of the TOS exposed to the different concentrations of sucrose (*n* = 3). h) Classification of the signals based on PCA. Data are presented as Mean ± SD. * indicates *p* < 0.05. ** indicates *p* < 0.01. *** indicates *p* < 0.001.

To study the ability of the taste organoids‐on‐a‐chip system to recognise varying degrees of sweetness, the taste organoids were exposed to 1, 0.75, 0.5, 0.25, and 0.125 g mL^−1^ sucrose, while the taste organoids without sucrose stimulation were set as a control. Sweet substances can bind in the cleft of a venus flytrap module of T1R2 or T1R3, activate the G*α*‐gustducin pathway, lead to an increased secondary messenger level, and ultimately result in an action potential for sweet signal transduction^[^ ^25^ ^]^. The typical extracellular potentials of the taste organoids exposed to varying concentrations of sucrose and their energy distributions in 0–40 Hz were shown in **Figure** **5**a and Figure 5b. The radar map compared the normalized 8 parameters extracted from the extracellular potentials of the varying groups (Figure 5c). When taste organoids were exposed to 1 g mL^−1^ sucrose, the PSD of the *δ*, *θ*, *α*, or *β* wave was significantly higher than that of the control group, despite no obvious change being shown in the PSD of the *γ* wave (Figure 5d). When the concentration of sucrose went down from 1 to 0.75 g mL^−1^, the PSDs of *δ*, *θ*, and *α* waves were not obviously changed, but the PSDs of *β* and *γ* waves significantly increased. When taste organoids were exposed to sucrose lower than 0.75 g mL^−1^, the PSDs of all five waves gradually declined with the decrease of the sucrose concentration. No significant difference was shown between the 0.125 g mL^−1^ sucrose group and the control group in the PSDs of all five waves. Taken together, 0.25‐1 g mL^−1^ sucrose could rise the PSDs of *δ*, *θ*, and *α* waves, but only 1, 0.75 and 0.5 g mL^−1^ sucrose rose the PSDs of *β* waves, while only 0.75 and 0.5 g mL^−1^ rose PSDs of *γ* waves.

We next analyzed the effects of different concentrations of sucrose on the fire rate, maximum amplitude, and average amplitude of spikes in the extracellular potentials of the taste organoids. In general, all these three parameters first increased and then decreased with the decrease of the sucrose concentration from 1 to 0.25 g mL^−1^ (Figure 5e,f). When the sucrose concentration was lower than 0.25 g mL^−1^, all three parameters had no significant difference from the ones of the control group. The fire rate of spikes in the extracellular potentials reached its peak at the sucrose concentration of 0.75 g mL^−1^ (Figure 5e), while the maximum amplitude reached its peak at the sucrose concentration of 0.5 g mL^−1^ (Figure 5f). Similar to the fire rate, the average amplitude of spikes in the extracellular potentials reached its peak at the sucrose concentration of 0.75 g mL^−1^, but no significant difference was shown in the numbers of the average amplitude between 0.75 and 0.5 g mL^−1^ sucrose group (Figure 5g). PCA data showed that the extracellular potential signals of taste organoids in the 6 groups were clustered into 5 types (Figure 5h). Among the 6 groups, 0.125 g mL^−1^ of sucrose and the control group were clustered into one type, suggesting that the taste organoids‐on‐a‐chip system was not able to recognize the sweetness of sucrose lower than 0.125 g mL^−1^. Altogether, these results indicated that the taste organoids‐on‐a‐chip system was functioning in recognizing varying degrees of sweetness.

### 2.7 Recognition of Bitterness Using the Taste Organoids‐on‐a‐Chip

**Figure 6:**
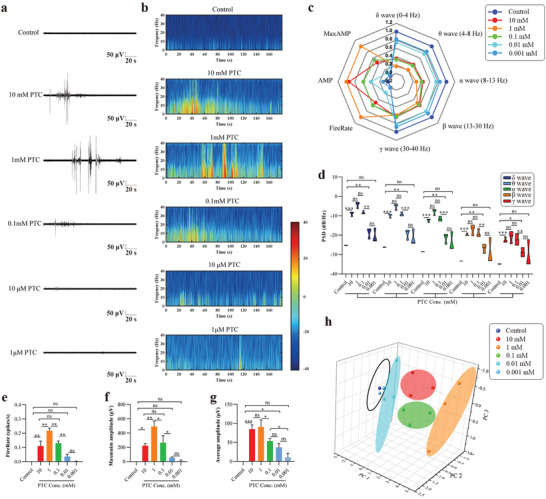
Response of taste organoids‐on‐a‐chip to PTC. a) Representative signals of the TOS exposed to 10, 1, 0.1, 0.01, and 0.001 mm PTC. b) Joint time‐frequency analysis of the representative signals. c) Differences in the 8 parameters among the signals of the TOS exposed to the different concentrations of PTC. d) Comparison of PSD of different frequency bands among the signals of the TOS exposed to the different concentrations of PTC (*n* = 3). e) Fire rates of spikes in the signals of the TOS exposed to the different concentrations of PTC (*n* = 3). f) The maximum amplitudes of spikes in the signals of the TOS exposed to the different concentrations of PTC (*n* = 3). g) The average amplitudes of spikes in the signals of the TOS exposed to the different concentrations of PTC (*n* = 3). h) Classification of the signals based on PCA. Data are presented as Mean ± SD. * indicates *p* < 0.05. ** indicates *p* < 0.01. *** indicates *p* < 0.001.

To study the ability of the TOS to recognise varying degrees of bitterness, the taste organoids were exposed to 10, 1, 0.1, 0.01, and 0.001 mm PTC, while the taste organoids without PTC stimulation were set as a control. Cellular bitter signal transduction shares similar signal pathways with sweet signal transduction and can finally lead to depolarization of the cell membrane.^[^ ^4b^ ^]^ However, the taste receptors for these two signal transduction are different and always expressed in different type II cells.^[^ ^3b^ ^]^ Indeed, cells which were sensitive to sucrose were found insensitive to PTC and vice versa (Figure 2c). The typical extracellular potentials of the taste organoids exposed to varying concentrations of PTC and their energy distributions in 0–40 Hz were shown in **Figure** **6**a and Figure 6b. The radar map compared the normalized 8 parameters extracted from the extracellular potentials of the varying groups (Figure 6c). Compared to the control group, noticeable higher PSDs of the *δ*, *θ*, *α*, *β*, and *γ* waves were found in the groups where the concentration of PTC are higher than 0.01 mm (Figure 6d). Among the 10, 1, and 0.1 mm PTC groups, no significant difference was shown in the PSDs of all 5 waves. However, when the concentration of PTC declined from 0.1 to 0.01 mm, drops were observed in all 5 waves. In addition, there was no significant difference in PSDs of the 5 waves between the 0.01 and 0.001 mm PTC group. These results indicated that the PTC changed the PSDs of the taste organoids in all five waves and the changes in the five waves had a similar tendency.

Meanwhile, we analyzed the differences in fire rates, maximum amplitudes and average amplitudes of spikes that arose from the different PTC concentrations. The fire rate and the maximum amplitude raised first, reached their peaks at 1 mm, and then declined with the decrease of the PTC concentration (Figure 6e,f). No significant difference was shown between 10 and 0.1 mm in fire rate and the maximum amplitude. For the average amplitude, no significant change was observed when the PTC concentration declined from 10 to 1 mm, but then the average amplitude went down gradually with the decline of the PTC concentration (Figure 6g). In addition, the fire rates, maximum amplitudes and average amplitudes were significantly larger than those of the control group when the PTC concentration was higher than 0.1 mm (Figure 6e–g). Although no significant difference in the fire rate and the maximum amplitude was shown between the 0.01 mm PTC group and the control group, the average amplitude of the 0.01 mm PTC group was significantly higher than that of the control group. Based on the PCA results, the extracellular potentials of the taste organoids in all 5 PTC groups and the control group were divided into 5 types (Figure 6h). In particular, the extracellular potentials in the 0.001 mm PTC group and the control group were clustered into one type, suggesting that the taste organoids‐on‐a‐chip system was not able to recognize the bitterness of PTC lower than 0.001 mm. Taken together, these results demonstrated that the TOS had a function in recognizing varying degrees of bitterness.

### 2.8 Recognition of Saltiness Using the Taste Organoids‐on‐a‐Chip

**Figure 7:**
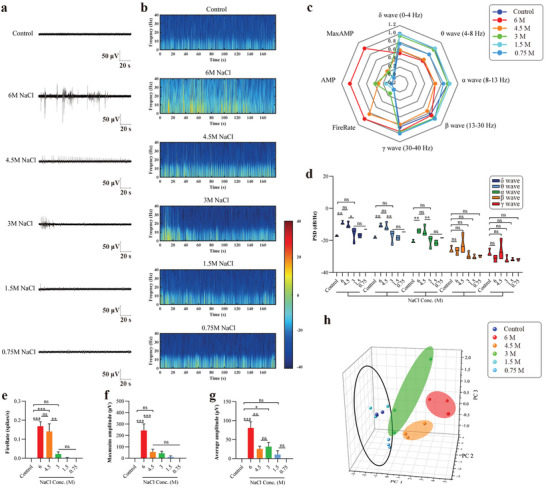
Response of taste organoids‐on‐a‐chip to NaCl. a) Representative signals of the TOS exposed to 6, 4.5, 3, 1.5, and 0.75 m NaCl. b) Joint time‐frequency analysis of the representative signals. c) Differences in the 8 parameters among the signals of the TOS exposed to the different concentrations of NaCl. d) Comparison of PSD of different frequency bands among the signals of the TOS exposed to the different concentrations of NaCl (*n* = 3). e) Fire rates of spikes in the signals of the TOS exposed to the different concentrations of NaCl (*n* = 3). f) The maximum amplitudes of spikes in the signals of the TOS exposed to the different concentrations of NaCl (*n* = 3). g) The average amplitudes of spikes in the signals of the TOS exposed to the different concentrations of NaCl (*n* = 3). h) Classification of the signals based on PCA. Data are presented as Mean ± SD. * indicates *p* < 0.05. ** indicates *p* < 0.01. *** indicates *p* < 0.001.

To study the capability of the taste organoids‐on‐a‐chip system to recognise varying degrees of saltiness, the taste organoids were exposed to 6, 4.5, 3, 1.5, and 0.75 m NaCl, while the taste organoids without NaCl stimulation were set as a control. It is still unclear exactly which taste bud cells transduce NaCl, let alone how the transduction was performed in the cells.^[^ ^5^ ^]^ However, studies have shown that sodium salts, such as NaCl, NaAc, and NaGlu, can lead to the depolarization of some taste bud cells.^[^ ^26^ ^]^ The taste bud cells that are sensitive to NaCl were found in the taste organoids and the typical extracellular potentials of the taste organoids exposed to varying concentrations of NaCl were shown in **Figure** **7**a. The energy distributions of the typical extracellular potentials in 0–40 Hz were visualized in Figure 7b. Differences in the 8 normalized parameters among varying concentrations of NaCl are shown in the radar map (Figure 7c). There was no significant difference in both *β* and *γ* waves between the control group and varying concentrations of NaCl groups (Figure 7d). For *δ*, *θ*, and *α* waves, no significant change was observed until the concentration of NaCl went up to 4.5 m compared to the control group, but no significant difference was shown between 4.5 and 6 m (Figure 7d).

Similarly, for fire rate, no significant change was found until the concentration of NaCl went up to 4.5 m compared to the control group and no significant difference was shown between 4.5 and 6 m (Figure 7e). For the maximum amplitude, a significant difference was only shown between 6 m and the control (Figure 7f). For average amplitude, the values went up with the increase of the concentration of NaCl and they were significantly larger than the control when the concentration of NaCl was higher than 3 m (Figure 7g). No significant difference was observed between 3 and 4.5 m in average amplitude (Figure 7h). All the extracellular potentials in the 6 groups were clustered into 4 types using the PCA method. In particular, the extracellular potentials from 0.75 and 1.5 m of NaCl groups and the control group were considered as one type, demonstrating that the TOS was not able to recognize the saltiness of NaCl lower than 1.5 m. Altogether, these results indicated that the taste organoids‐on‐a‐chip system was functioning in recognizing varying degrees of saltiness.

In this study, the taste thresholds of the TOS for acetic acid, PTC, NaCl, and sucrose were 1.56 mg mL^−1^, 0.01 mm (1.52 µg mL^−1^), 3 m (0.18 g mL^−1^), and 0.25 g mL^−1^, respectively. According to previous studies, the taste threshold of acetic acid, sucrose, PTC, and NaCl for C57 mice was 0.125 mg mL^−1^, 0.05 g mL^−1^, 0.19 mg mL^−1^, and 2 g mL^−1^, respectively.^[^ ^27^ ^]^ The differences may be a result of the different expression of taste receptors in taste receptor cells caused by different culture conditions (Figure 1d).

## 3 Discussion

With the rapid growth in the food industry, there is an urgent need for developing bioelectronic tongues that can mimic the biological functions of the human gustatory system for evaluating the qualities of food or beverage. Inspired by the mammalian gustatory system, we developed the taste organoids‐on‐a‐chip system to highly mimic the mammalian taste perception in vitro. Compared to traditional ETs and non‐cell biorecognition elements based ETs, the TOS is able to reflect real taste information. In the TOS, the microelectrodes functioning as gustatory axons were coupled with the taste organoids to receive gustatory information, mimicking the taste bud structure (Figure 2a). Similar to in vivo conditions where basic tastes were sensed by different taste receptor cells in taste buds and mediated by different neurons,^[^ ^5^, ^7^ ^]^ different taste information were generated by different taste receptor cells in taste organoids and were transmitted by distinct microelectrodes in the TOS (Figure 2c). In addition, the taste organoid composed of multiple cell types of their tissue of origin can somehow recreate the in vivo taste perception environment in both physiological and pathophysiological situations. Therefore, our bioelectronic tongue can reflect the real taste information from mammalian perspectives to a certain degree.

Compared to lingual epithelium or lingual epithelium derived primary cells based ETs, the TOS is more stable, and the taste recognition is more repeatable. Taste stem/progenitor cells in the on‐chip taste organoids are proliferable and can differentiate into different taste receptor cells, which helped the taste organoid maintain sensing functions and high cell viability (Figure 1b–d and Figure 3e–g) and potentially increase the life span of the TOS. In addition, the taste organoids used for constructing the bioelectronic tongue are reproducible, which means different chips can be made by the organoids derived from the same mice. This ability avoids the TOS from the effects of individual variabilities and increases the repeatability of taste recognition.

The taste recognition of varying concentrations of taste stimuli in this study can be achieved through a simple analysis method based on signal feature extraction and PCA. Through this method, the taste threshold reached 1.56 mg mL^−1^, 0.25 g mL^−1^, 0.01 mmol L^−1^, and 3 mol L^−1^ for acetic acid, sucrose, PTC, and NaCl, respectively (Figures 4, 5, 6, 7). However, the accuracy and range of detection can potentially be further improved by deep learning. Deep learning has dramatically improved the state‐of‐the‐art (SOTA) in speech recognition, visual object recognition, object detection and many other domains such as drug discovery and genomics.^[^ ^28^ ^]^ For example, H. Wang et al. developed a deep learning method based on an end‐to‐end trainable network that mines implicit contextual knowledge behind scene text image and enhance the semantics and correlation to fine‐tune the image representation, which outperformed the SOTA by 3.72% and 5.39%.^[^ ^29^ ^]^ Q. Song et al. proposed a deep learning method with multimodal sparse transformer network (MMST) and achieved a better performance (≈5% lower word error rate compared to SOTA) for different types of noise (−5 to 10 dB).^[^ ^30^ ^]^ In addition to these, many studies achieved high accuracy and wide range of detection by using deep learning.^[^ ^31^ ^]^ Therefore, the deep learning is hopeful to further increase the performance of the TOS in taste recognition in the future.

The TOS is expected to be used in food quality controls, disease modelling, and drug screening. With the SARS‐CoV‐2 spreading worldwide and new variants of the virus continuing to emerge, COVID‐19‐related complications, including gustatory dysfunction, have received considerable attention.^[^ ^32^ ^]^ Although multiple organoid models have been used for studies of SARS‐CoV‐2 infection, such as respiratory organoids and intestinal organoids,^[^ ^32e^ ^]^ there's a lack of effective in vitro models for studies of gustatory dysfunction arose by SARS‐CoV‐2. The TOS can be used in the studies of COVID‐19‐related gustatory dysfunction by infecting the taste organoids with SARS‐CoV‐2. Apart from viruses, gustatory dysfunction can be caused by other factors, such as medicines, ageing, and glossodynia,^[^ ^33^ ^]^ which can also be potentially modeled using the TOS for pathogenesis exploration and drug development. In addition, TOS can also be used in the evaluation of drug side effects. Taste dysfunction arising from chemotherapy in cancer patients impacts the quality of life and impairs oral intake, which may have broader implications consisting of weight loss and nutritional compromise.^[^ ^34^ ^]^ These consequences may in turn affect broad symptom clusters including tissue healing, energy levels, and mood.^[^ ^35^ ^]^ However, knowledge about how to investigate this side effect of chemotherapeutic drugs preclinically is scarce.^[^ ^36^ ^]^ Based on the ability to quantify taste perception, TOS may be a personalized solution for the preclinical evaluation of the impact of the drugs on taste function. Moreover, TOS can potentially be used as a biosensor in the food industry, such as the wine industry. The quality of wines is usually evaluated by a sensory panel formed of trained experts and instruments, such as FTIR spectra, mass spectrometry, and an array of sensors.^[^ ^37^ ^]^ However, these strategies are expensive and time‐consuming or cannot obtain the real taste of the wine.^[^ ^38^ ^]^ Using taste organoids as sensory receptors, TOS could be used to describe the wine from the human perspective and replace mankind to some extent.

As a proof of concept, we developed the TOS mainly for ex vivo use. In the future, the TOS is expected to be adapted to living creatures. In this condition, the toxicity of the taste organoids‐on‐a‐chip and the portability of the whole system should be taken into consideration. Therefore, the device should be as small as possible to reduce foreign body sensation and made of non‐toxic materials. In addition, the taste organoids‐on‐a‐chip should be made by flexible electrodes to avoid injury to the surrounding tissue and the signal acquisition and transmission devices should be portable. By far, the MEA chip and the signal acquisition and transmission devices used can hardly meet the requirements. Existing wearable electronic organoid devices^[^ ^39^ ^]^ set examples for how to adapt the TOS to human bodies. Our ongoing research is carrying on to adapt our bioelectronic tongue to the living body by using flexible electrodes and portable signal acquisition and transmission devices.

## 4 Conclusion

In this study, we developed a taste organoids‐on‐a‐chip system where taste organoids were used as recognition elements, a microelectrode array chip was used for real‐time recording of the extracellular potential of the taste organoids, and a computer was used for signal processing. The taste organoids maintained key taste receptors expression even after the third passage and maintained high cell viability during 7 days of on‐chip culture. In addition, the TOS distinguished sour, sweet, bitter, and salty stimuli with great specificity and recognized varying concentrations of acetic acid, sucrose, PTC, and salty.

## 5 Experimental Section

### Cells Isolation

To obtain single cells for taste organoid culture, tongues were cut out from sacrificed C57 mice (3 weeks old, Zhejiang Laboratory Animal Center) and then injected with an enzyme mixture containing 0.025% trypsin (Gibco) and collagenase (1 mg mL^−1^, Roche) for 30 min at 37 °C. After that, the epithelium was gently peeled off. Then, the circumvallate papilla was dissected out from the epithelium and treated with 0.25% trypsin at 37 °C for 2 h. A pipette was used to dissociate the circumvallate papillae into signal cells and a 70‐µm nylon mesh was used for filtering the undissociated tissue. The result cell suspension was centrifuged at 300 × g for 3 min and the cells were resuspended in Advanced DMEM/F12 medium (Gibco) at a cell density of 10^6^ cells per mL for future use. The animal study was approved by the Animal Ethics Committee of Zhejiang University (ZJU20200078).

### Taste Organoid Culture

For taste organoid culture, single cells were mixed with chilled Matrigel (growth factor reduced, Corning) at a volume ratio of 1:2. A volume of 60 µL mixture was seeded on each well of a pre‐warmed 24‐well plate. Then, the plate was put into the incubator at 37 °C for 20 min. After that, 1 mL Advanced DMEM/F12 medium supplemented with 1% N2 (Life Technologies), 2% B27 (Life Technologies), 200 ng mL^−1^ R‐spondin‐1 (R&D), 100 ng mL^−1^ Noggin (PeproTech), 1 µm Jagged‐1 (Topscience), 10 µm Y27632 (Aladdin), 1 mm N‐acetylcysteine (Sigma), and 50 ng mL^−1^ mouse epidermal growth factor (Life Technologies) was added into each well; The recipe for the medium cocktail was from a previous study^[^ ^16^ ^]^. The cells were cultured in the incubator supplemented with 5% CO_2_ at 37 °C and the culture medium was changed every other day. For organoid passage, the culture medium was withdrawn and 1 mL cell recovery solution (Corning) was used to recover the organoids following the manufacturer's instructions. The recovered organoids were digested into single cells using 0.25% trypsin.

### Immunostaining

For immunostaining, organoids were recovered from Matrigel and then fixed in 4% paraformaldehyde overnight. The fixed organoids were then treated with 0.3% Triton X‐100 for 30 min. After washing with 1 × PBS 3 times, the organoids were blocked by 5% BSA at 37 °C for 1.5 h. To mark taste receptors in the organoid, rabbit anti‐OTOP1 (Antibodies‐online, ABIN7075125) and pig anti‐TAS1R2 (NOVUS, NBP2‐80499) were used as primary antibodies, while goat anti‐rabbit IgG (Abcam, ab150077) and goat anti‐pig IgG (Abcam, ab150187) were used as secondary antibodies, respectively. All primary antibodies were diluted 300 times and all secondary antibodies were diluted 500 times. Both stainings using primary antibodies and secondary antibodies were followed by washing with 1 × PBS 3 times. The nucleus was stained using a DAPI solution (Solarbio). An Olympus FV3000 confocal microscope was used to acquire images.

### Quantitive Reverse Transcription PCR (RT‐qPCR)

For RT‐qPCR, a MiniBEST Universal RNA Extraction Kit (Takara) was used to extract total RNA in organoids or circumvallate papillae following the manufacturer's instructions. Then, a PrimeScript™ RT reagent Kit with gDNA Eraser (Takara) was used to reverse transcript mRNA of the total RNA to cDNA. An amount of cDNA equivalent to 1pg to 100 ng of total RNA (2 µL) was mixed with 1 µL 10 µm forward primer, 1 µL 10 µm reverse primer, 6 µL nuclease‐free water, and 10 µL iQ™ SYBR Green Supermix (2×concentration, Bio‐Rad) for real‐time PCR set up. The primers used were synthesized by Sangon Biotech and are listed in Table S1 (Supporting Information). A CFX Connect™ Real‐Time System (Bio‐Rad) was used to run the qPCR. The data acquired was processed by a 2^−ΔΔCt^ method according to previous work.^[^ ^17^ ^]^

### Set‐Up of the Bioelectronic Tongue System

The bioelectronic tongue system was composed of a taste organoids‐on‐a‐chip, a MEA2100‐System (Multichannelsystems), a cell culture incubator, and a computer. The taste organoids‐on‐a‐chip was made by coupling taste organoids with a MEA chip. The MEA chips were fabricated and microelectrodes of the chips were electroplated with platinum black according to previous work.^[^ ^18^ ^]^ For fabrication of taste organoids‐on‐a‐chip, taste organoids were harvested on day 14 and put onto microelectrodes of the MEA chip overnight in the medium. After that, the medium was withdrawn and a volume of 3 µL Matrigel was then gently pipetted from the top to where the organoids were located, followed by a 20‐min curing process in the incubator at 37  °C. For the construction of the bioelectronic tongue system, 1 mL medium was added to the taste organoids‐on‐a‐chip and the chip was put on the MEA2100‐System which was connected to the computer. The cell culture incubator was used to maintain an essential environment (37°C, 5% CO_2_) for taste organoids‐on‐a‐chip maintenance. For recognition of the tastants, the medium in the chip was replaced by the medium containing the taste stimulus and the extracellular changes were recorded for 3 min. After that, the medium was moved out and the chip was washed 3 times with 1 × PBS. Then, the chip was refilled with 1 mL culture medium and placed in the incubator for at least 20 min to recover the gustatory sensitivity.

### Live/Dead Staining

For live and dead staining, 500 organoids harvested on day 14 were seeded on each chip or well of 24‐well plates. After 1, 3, 5, or 7 days of culture, the organoids were directly stained or digested into single cells using 0.25% trypsin for further staining. A Calcein‐AM kit (Dojindo) was used to label live cells and a PI kit (Dojindo) was used to label dead cells following the manufacturer's instructions. An inverted fluorescence microscope (Nexcope) was used to visualize the organoids or cells and the ImageJ software was used to count live or dead cells. The cell viability equals the number of living cells divided by the total number of cells multiplied by 100%.

### Lactate Dehydrogenase Cytotoxicity Assay

For LDH extracting, a number of 500 organoids harvested on day 14 were seeded on each chip or well of 24‐well plates. After 1, 3, 5, or 7 days of culture, the culture medium was withdrawn from the chip or the well and centrifuged at 400 × g for 5 min. The deposit was then resuspended in 1 mL LDH releasing reagent and the resulting mixture was pipetted back to the chip or the well. Then, the chip or the plate was incubated at 37°C for 1 h for LDH release. After that, the mixture was centrifuged at 400 × g for 5 min and the resulting supernatant was used for the measurement of LDH enzyme activity. An LDH cytotoxicity assay kit was used to measure the LDH release in organoids on the chips compared to 24‐well plates following the manufacturer's instructions. A fluorescence microplate reader (SpectraMax Paradigm, Molecular Devices) was used to measure the absorbance at 490 nm.

### Signal Processing

For signal processing, the extracellular potential signals were analyzed in both frequency and time domains using MATLAB (R2019b, The MathWorks, U.S.A.) and Python software. To characterise the frequency domain of the signals, spectrogram analysis was carried out. The spectrogram was obtained using the short‐time Fourier transform where the Hamming window was performed. Then the average energy values of the five frequency bands (*δ*(0‐4 Hz), *θ*(4‐8 Hz), *α*(8‐12 Hz), *β*(12‐30 Hz), and *γ*(30‐40 Hz)) were calculated based on the spectrogram results. The power spectral densities of the five frequency bands were calculated according to the previous study.^[^ ^19^ ^]^ For the characterization of signals in the time domain, the fire rate, the maximum amplitude (peak‐to‐peak value) of spikes, and the average amplitude of spikes were analyzed. A threshold of 3‐times standard deviation was set to filter out the noise data. Finally, the obtained three time‐domain features and five frequency‐domain features were subjected to dimensionality reduction using the principal component analysis (PCA), and the resultant three principal components were visualized in three dimensions.

### Data Analysis

GraphPad Prism 8 (GraphPad Software Inc., USA) was used for data analysis. Ordinary one‐way ANOVA was used for multiple comparisons, while uncorrected Fisher's LSD was used to calculate P‐value.

## Conflict of Interest

The authors declare no conflict of interest.

## Supporting information

Supporting Information

## Data Availability

The data that support the findings of this study are available from the corresponding author upon reasonable request.
