# Supplementary material for: Mimicking the Biological Sense of Taste In Vitro Using a Taste Organoids‐on‐a‐Chip System
Source: Adv Sci (Weinh). 2023 Jan 13;10(7):2206101. doi: 10.1002/advs.202206101 (PMC9982573; doi:10.1002/advs.202206101)
Supplement: Supplementary file 1 — Supporting Information [file ADVS-10-2206101-s001.pdf]

Supporting Information

Mimicking the biological sense of taste in vitro using a taste organoids-on-a-chip system

Jianguo Wu [1, 2, 3, #], Changming Chen [1, 2, #], Chunlian Qin [1, 2], Yihong Li [4], Nan Jiang [1, 2], Qunchen Yuan [1, 2], Yan Duan [1, 2], Mengxue Liu [1, 2], Xinwei Wei [1, 2], Yiqun Yu [5], Liujing Zhuang [1, 3, *], Ping Wang [1, 2, 3, 6, *]

[1] *Biosensor National Special Laboratory, Key Laboratory for Biomedical Engineering of Education Ministry, Department of Biomedical Engineering, Zhejiang University, Hangzhou 310027, China*

[2] *The MOE Frontier Science Center for Brain Science and Brain-Machine Integration, Zhejiang University, Hangzhou 310027, China*

[3] *State Key Laboratory of Transducer Technology, Chinese Academy of Sciences, Shanghai 200050, China*

[4] *College of Life Sciences, Zhejiang University, Hangzhou 310058, China*

[5] *Department of Otolaryngology, Eye, Ear, Nose and Throat Hospital, Shanghai Key Clinical Disciplines of Otorhinolaryngology, Fudan University, Shanghai, China 200031.*

[6] *Cancer Center, Zhejiang University, Hangzhou 310058, China*

#: These authors contributed equally.

[*]Corresponding author: Liujing Zhuang (thisiszlj@163.com); Ping Wang (cnpwang@zju.edu.cn);

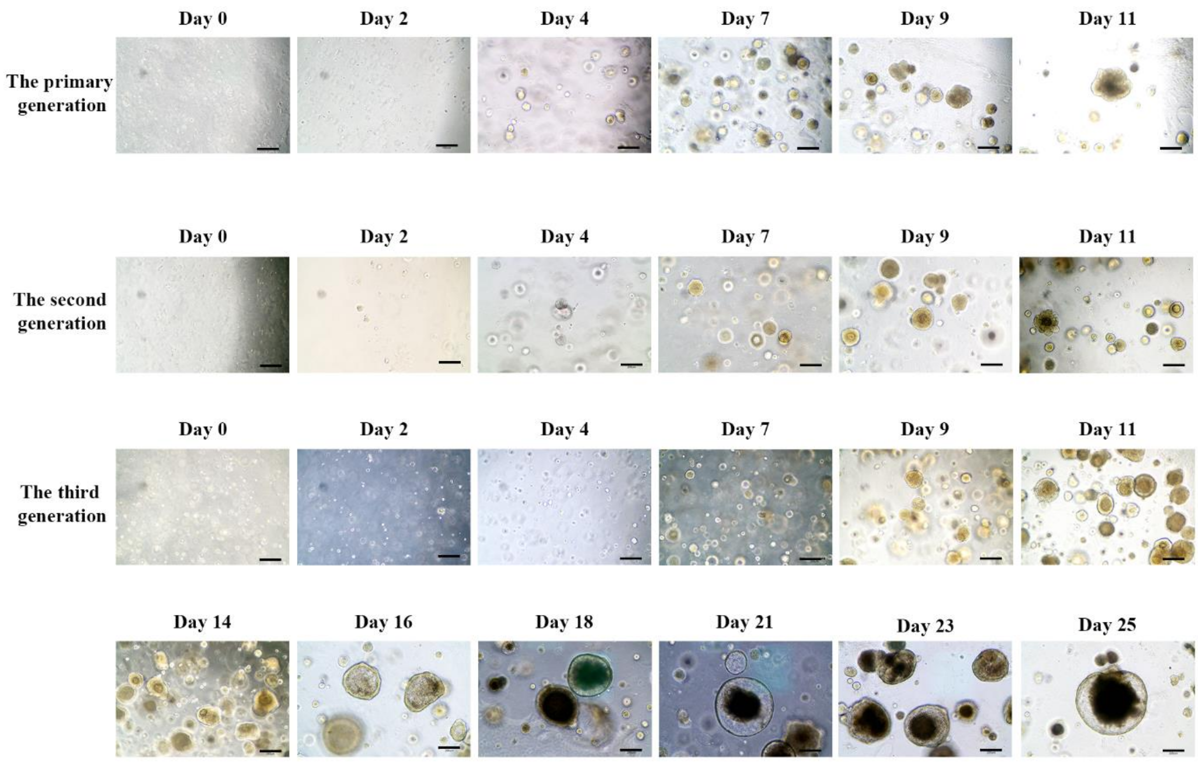

Figure S1. Bright field images of taste organoids during the culture process.

Table S1. Primers of T2Rs for RT-qPCR

Gene	Sequence	
T2R102	Forward primer	5'-CTC CTG CTA ATC TTC TCT TTG TG-3'
	Reverse primer	5'-GGG TCT CTG TGT CTT CTG G-3'
T2R103	Forward primer	5'-AGC ACA GTG GCC CAC ATA AA-3'
	Reverse primer	5'-TGG CCT GTG GGA AAA GCT AC-3'
T2R104	Forward primer	5'-GCA ACA CAT CCT GGC TGA T-3'
	Reverse primer	5'-CCC CAT ATT GGC AAA AAC AT-3'
T2R105	Forward primer	5'-AAG GCA TCC TCC TTT CCA TT-3'
	Reverse primer	5'-GTG CAA TAA ATG TGT TCC CTA AAA-3'
T2R106	Forward primer	5'-AGC CAC ATT CTT CTC AAC CT-3'
	Reverse primer	5'-AGC ATG TAA TGA TAG CCA CCA-3'
T2R107	Forward primer	5'-GGC ATC CTC CTT TGT GTT GT-3'
	Reverse primer	5'-TGC AAT ATA TGT GTC CCC TAA AAC-3'
T2R108	Forward primer	5'-GTT TCT CCT GTT GAA ACG GAC T-3'
	Reverse primer	5'-GTG AGG GCT GAA ATC AGA AGA-3'
T2R109	Forward primer	5'-GTC AAA TTC AGG TGT TAG GAA GTC-3'
	Reverse primer	5'-CAC AGG GAG AAG ATG AGC AG-3'
T2R110	Forward primer	5'-CTT TCT CAT GCT CAT CTT CTC AC-3'
	Reverse primer	5'-GGC ATC TCT AGG TGG TTT GG-3'
T2R113	Forward primer	5'-CCA CGG TAA TGT TTT CTT TGC-3'
	Reverse primer	5'-TGG TGC TGA TGT CTC TGC AT-3'
T2R114	Forward primer	5'-CGG CTG CCA CTC ACT TAT C-3'
	Reverse primer	5'-CAG CAC TTT AAT AGT TGC AGT ATC ATT-3'
T2R115	Forward primer	5'-CCT TTG GTG TAT CCT TGA TAG CTT-3'
	Reverse primer	5'-CTG CAT CTT CCT TAC ATG TTT CA-3'
T2R116	Forward primer	5'-AAG GTT TGG AGT GCT CTG CT-3'
	Reverse primer	5'-AGC TGT TCT TGC AAC CTG TGT-3'
T2R117	Forward primer	5'-CCC TGT GGA CAC ATC ACA AG-3'
	Reverse primer	5'-TCA CAG TTT GTA GGG CTT TGA A-3'
T2R118	Forward primer	5'-CAC TGG GTG CAG ATG AAA CA-3'
	Reverse primer	5'-CTT CAG AAC AGT GAA CTG AGC TTT-3'
T2R119	Forward primer	5'-AAG GAA CCC AAG ACT CAG TGA C-3'
	Reverse primer	5'-AGG CTT CTG AGC AGG ATG TC-3'
T2R120	Forward primer	5'-TGT TAA CGA ACT GGC ATT CAC-3'
	Reverse primer	5'-GGT TGG TTA TAG CCC AGG T-3'
T2R121	Forward primer	5'-CTG GTC TTA TTG GAG ATG ATT GTG-3'
	Reverse primer	5'-GGA GAA GAT TAA CAG GAT GAA GGA-3'
T2R122	Forward primer	5'-TCT TCT CTT TAT GGA GCC ACC T-3'
	Reverse primer	5'-GCT TCT GTG CTT ATG TCT TTG G-3'
T2R123	Forward primer	5'-CAT TAA AGC CTT GCA AAC TGT G-3'
	Reverse primer	5'-GGA AAA GTA AGT ATA TGG CAT ACA GCA-3'
T2R124	Forward primer	5'-CTA CGG CCC ACA GAA ATG CC-3'
	Reverse primer	5'-AGC TGC CTC ATT ACC CAA AGA-3'

Gene	Sequence	
T2R125	Forward primer	5’-AAG GCC TTG CAC ATG GTA GT-3’
	Reverse primer	5’-GGC AAG AGA CAA AAA GAA AAC TG-3’
T2R126	Forward primer	5’-GTG TGT GGG ATT GGT CAA CA -3’
	Reverse primer	5’-GCT CCC GGA GTA CTC AAC C-3’
T2R129	Forward primer	5’-TTT AGC ATG TGG CTT GCT GC-3’
	Reverse primer	5’-AGA GGC CCA AAG ACA TGA GC-3’
T2R130	Forward primer	5’-TGC ATT CAT TGC ACT GGT AAA -3’
	Reverse primer	5’-GAT TAA ATC AAT AGA GGC AAT CTT CC-3’
T2R131	Forward primer	5’-TAG CCC ACA TTT CCC ATC C-3’
	Reverse primer	5’-CAA GCA CAC CTC TCA ATC TCC-3’
T2R134	Forward primer	5’-GCC TGG GAA GTG GTA ACC TA -3’
	Reverse primer	5’-GTT GCT TAG TAT CAG AAT GGT GGA-3’
T2R135	Forward primer	5’-CCA TCA TGT CCA CAG GAG AA-3’
	Reverse primer	5’-TCA GTA GTC TGA CAT CCA AGA ACT GT-3’
T2R136	Forward primer	5’-GGA CAA TGA GGC TTT ATG GAA-3’
	Reverse primer	5’-CCT TAA TGT GGG TTG AAG CAC-3’
T2R137	Forward primer	5’-CTG GCT CAA ATG GAG AGC TT-3’
	Reverse primer	5’-GGT ACT GAC ACA GGA TAA GAG CAG-3’
T2R138	Forward primer	5’-CAA ACC AAG TGA GCC TCT GG-3’
	Reverse primer	5’-GAG AAG CGG ACA ATC TTG GA-3’
T2R139	Forward primer	5’-ATG GCT CAA CCC AGC AAC TAC-3’
	Reverse primer	5’-ACA GCC ATG ACA ATC CCA CT-3’
T2R140	Forward primer	5’-GAA GAA CAT GCA ACA CAA TGC-3’
	Reverse primer	5’-AGG GCC TTA ATA TGG GCT GT-3’
T2R143	Forward primer	5’-CAT TGG CCT CTA TGT TGC AG-3’
	Reverse primer	5’-TGT CCG GTT CCT CAT CCA-3’
T2R144	Forward primer	5’-AAG CAG AAA ATC ATA GGG CTG A-3’
	Reverse primer	5’-TGA AGG AAA CCA ACA CTG ACA-3’
β-actin	Forward primer	5’-GCA GGA GTA CGA TGA GTC CG-3’
	Reverse primer	5’-ACG CAG CTC AGT AAC AGT CC-3’
